# Inventory study on completeness of tuberculosis case notifications in Poland in 2018

**DOI:** 10.2807/1560-7917.ES.2024.29.1.2300081

**Published:** 2024-01-04

**Authors:** Teresa Domaszewska, Maria Korzeniewska-Kosela, Barbara Hauer, Nita Perumal, Stefan Wesolowski, Walter Haas, Stefan Kroeger

**Affiliations:** 1 Robert Koch Institute, Berlin, Germany; 2 National Tuberculosis and Lung Diseases Research Institute, Warsaw, Poland

**Keywords:** Tuberculosis, underreporting, inventory study, Poland, double-pronged

## Abstract

**Background:**

Evaluating tuberculosis (TB) notification completeness is important for monitoring TB surveillance systems, while estimating the TB disease burden is crucial for control strategies.

**Aim:**

We conducted an inventory study to assess TB reporting completeness in Poland in 2018.

**Methods:**

Using a double-pronged inventory approach, we compared notifications of culture-positive TB cases in the National TB Register to records of diagnostic laboratories. We calculated under-reporting both with observed and capture–recapture (CRC)-estimated case numbers. We further compared the notifications by region (i.e. voivodship), sex, and age to aggregated data from hospitalised TB patients, which provided an independent estimate of reporting completeness.

**Results:**

In 2018, 4,075 culture-positive TB cases were notified in Poland, with 3,789 linked to laboratory records. Laboratories reported further 534 TB patients, of whom 456 were linked to notifications from 2017 or 2019. Thus, 78 (534 – 456) cases were missing in the National TB Register, yielding an observed TB under-reporting of 1.9% (78/(4,075 + 78) × 100). CRC-modelled total number of cases in 2018 was 4,176, corresponding to 2.4% ((4,176 – 4,075)/4,176 × 100) under-reporting. Based on aggregated hospitalisation data from 13 of 16 total voivodeships, under-reporting was 5.1% (3,482/(3,670 – 3,482) × 100), similar in both sexes but varying between voivodeships and age groups.

**Conclusions:**

Our results suggest that the surveillance system captures ≥ 90% of estimated TB cases in Poland; thus, the notification rate is a good proxy for the diagnosed TB incidence in Poland. Reporting delays causing discrepancies between data sources could be improved by the planned change from a paper-based to a digital reporting system.

**Key public health message**

**What did you want to address in this study?**

Poland is a low incidence country for tuberculosis (TB), with 8.9 cases per 100,000 population notified in 2021. Evaluation of the under-reporting of TB cases in every country is important to provide more accurate estimates of the local TB incidence rates, assess efficacy of TB control strategies and monitor disease epidemiology and pathogen characteristics. We conducted an inventory study to assess TB reporting completeness in Poland in 2018.

**What have we learnt from this study?**

TB reporting completeness in Poland was found as ≥ 90% in 2018. This result suggested that the TB notification rate is a good proxy for the true TB incidence rate in Poland. Our study approach, based on the analysis of three datasets, was essential to assess TB reporting completeness despite limitations in data acquisition. Strengths of the Polish TB surveillance system and areas of possible improvement (notably notification timeliness) were identified.

**What are the implications of your findings for public health?**

Our analysis provides important insights into TB epidemiology in Poland, which can be used for national and international reporting. Together with Polish TB surveillance experts we developed suggestions to improve timely notifications of TB in the country. Successful implementation of our inventory study approach can inspire other countries in evaluating their disease surveillance systems, and further improving public health surveillance activities.

## Introduction

Tuberculosis (TB) continues to be a major global health threat, despite the identification of the causative mycobacteria from the group *Mycobacterium tuberculosis complex* over 140 years ago and increasing efforts dedicated to disease prevention and control. According to the World Health Organization (WHO), an estimated 10 million people got sick with TB and 1.5 million people died with TB in 2020 [1]. Despite the existing national surveillance systems and TB control strategies, there was a gap of an estimated 4.2 million TB patients who were either not diagnosed or not reported in 2020 [1]. Without efficient case-finding and complete case-reporting, it is difficult to appropriately reach out to affected people to treat them and to prevent further spread of infection, and consequently, to optimise national TB control strategies. The WHO Global Task Force on TB Impact Measurement, which was established in 2006, and which updated its mission in 2023, has been continuing to strengthen the routine surveillance of TB cases and deaths in all countries, as one of the strategic areas of work with the goal of reliably tracking TB epidemics in every country [2]. Work on strengthening surveillance included the development of a TB surveillance checklist of standards and benchmarks as well as guidance on digital recording and reporting. In countries, where TB is a notifiable disease, WHO defined ≥ 90% of TB reporting completeness as one of the benchmarks for a well-functioning TB surveillance system coverage [3]. In this respect, the recommendation to conduct inventory studies to measure under-reporting of detected cases, associated with support for their implementation, was emphasised. Between 2000 and 2021, 11 of 53 WHO European Region countries conducted and published results of inventory studies of TB registers: Croatia, Denmark, England, Finland, Germany, Italy, the Netherlands, Portugal, Romania, Slovenia, and Spain [4-11].

In Poland, which has a population of 38 million people distributed across 16 administrative units (voivodeships), 5,487 TB cases (including first diagnoses and people who had been treated in the past) were notified in 2018, corresponding to a TB incidence of 14.3 per 100,000 population per year [12,13]. TB incidence in Poland has been characterised by a decreasing trend since the beginning of the National TB Register (*Krajowy* *Rejestr* *Zachorowań na* Gruźlicę) in 1957 with a 5.2% decrease in 2018 compared with 2017 (5,487/5,787 cases) and 33.4% compared with 2009 (5,487/8,236 cases) [13]. In 2018, 74.3% of the notified cases were culture-positive (4,075/5,487 cases; denominator is skipped further in the text). Pulmonary TB constituted 95.2% of the notified TB cases in Poland (n = 5,224 cases). Most disease cases were reported in the 45–64-year age group (2,494 cases, corresponding to 45.5% of all cases) and cases among children below 15 years of age constituted 0.9% of the cases (n = 52 cases). The majority of the notified TB patients were male (n = 3,900 cases, 71.1%; 1,587 were female) and inhabitants of the cities were affected more frequently than inhabitants of villages (3,439 cases vs 2,048 cases, 62.7% vs 37.3%). Of all cases, 98.2% were detected among Polish citizens (n = 5,390 cases) [13]. WHO estimated the TB burden in Poland in 2018 as 6,000 cases (5,100–6,900; the lower and upper bounds are defined as the 2.5th and 97.5th centiles of outcome distributions produced in simulations) which corresponds to TB under-reporting rate of ca 9%. This estimate was produced using standard adjustment of notification rate for high-income countries [14,15]. However, no inventory study comparing national TB notification data to data of other independent healthcare providers (e.g. hospitals and laboratories) has been conducted so far.

Here, we investigate TB under-reporting among culture-positive cases notified in Poland in 2018 with a double-pronged inventory study approach. This methodology takes into consideration the Polish epidemiological situation, data availability, and surveillance system characteristics. Precisely, we compare the national case-based notification data of culture-positive cases in the National TB Register with the case-based data originating from diagnostic laboratories and with aggregated data on hospital admissions for TB. Since we observed considerable notification delays between the datasets while conducting the study, an additional analysis is dedicated to the quantification of the proportion of delayed notifications.

## Methods

**Figure 1:**
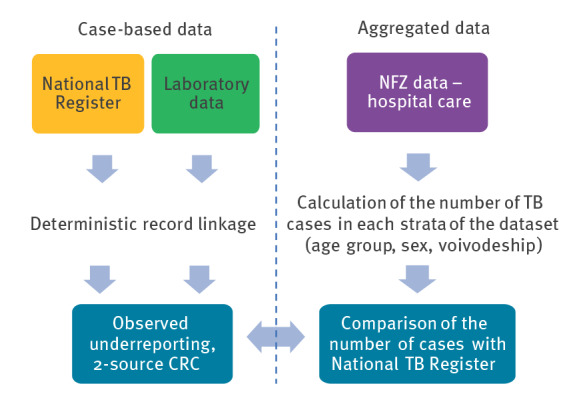
Flow chart of the two pathways within the double-pronged inventory approach, which is used to estimate under-reporting in the National Tuberculosis Register, Poland, 2018

CRC: capture–recapture analysis; NFZ: National Health Fund (national health insurance provider); TB: tuberculosis.

The double-pronged inventory study design implemented here was developed and used for the estimation of TB under-reporting in Germany [9], and is appropriate for the settings where: (i) a centralised, national TB notification register is kept, which is the case of Poland; (ii) only two independent case-based TB registers, containing records that can be linked, are available; in this study those are: the National TB Register (which contains national notification data), and the diagnostic laboratories data; (iii) additional independent aggregated TB register(s) are available; in this study, it is the inventory of hospital admissions of TB patients acquired from the National Health Fund of Poland (*Narodowy Fundusz Zdrowia*, NFZ); (iv) there are no sudden changes to TB epidemiology in the study time, which could disrupt reporting continuity; no such changes happened in Poland in 2017–2019. In a double-pronged inventory study design, a capture–recapture-analysis (CRC) using two case-based datasets (two-source CRC) is conducted alongside a parallel estimation of TB under-reporting using a third, independent, aggregated dataset − in this study, the NFZ dataset is aggregated by sex (collected as male, female or data missing), age, and voivodeship. The under-reporting estimates from both methods are subsequently presented and compared. All the steps of the study are described in detail below (Figure 1).

Sex is collected as male, female or data missing.

CRC methodology, stemming from ecology, allows modelling of the total population size. In epidemiology, it is implemented to estimate the completeness of disease registers [16]. The CRC relies on: closed population structure (assumption 1), independence of data sources (assumption 2), perfect matching across data sources (assumption 3), and the same probability of being included in the investigated datasets (assumption 4) [17]. Since CRC studies in epidemiology rarely meet all these assumptions, it is recommended to use at minimum three data sources to counteract the effect of the violation of assumptions on the study results [17]. Assumption 1 could not be entirely met in our study due to possible migrations and deaths of TB patients; however, it was assumed to have minimal influence on the CRC results, as migrant TB comprised less than 2% of Polish TB cases in 2018, and cases who died of TB or were diagnosed post-mortem are notified to the National TB Register. The datasets used for the study were considered independent (assumption 2), as laboratories and doctors separately and independently report the diagnosed TB cases to the Polish national Tuberculosis and Lung Diseases Research Institute (*Instytut Gruźlicy i Chorób Płuc*; IGiChP), which is the dedicated competent unit for TB surveillance and which maintains the National TB Register. Due to presence of the unique national personal identifier (PESEL) of patients in both laboratory and National TB Register reports, assumption 3 was met. Only culture-positive cases were included in the analysis to meet the criterion 4. Two-source CRC-analysis was implemented due to unavailability of a third case-based dataset.

### Data acquisition

TB prevention and control in Poland is regulated by law [18]. People diagnosed with TB must undergo compulsory hospitalisation during the infectious period or justified suspicion of infectious disease; treatment of pulmonary TB is compulsory. Physicians and feldshers (medical practitioners entitled to diagnose TB) who suspect or diagnose TB disease or death due to TB are required to report it within 24 hours to the competent state sanitary inspector. All case reports with detailed data on the TB patient, including patient’s name, sex, PESEL, home address, and diagnosis date are quarterly submitted to IGiChP, which holds the National TB Register. The National TB Register is the first case-based dataset used in this study, with its completeness investigated by comparison with other data sources. It is the official dataset used for national and international reporting and serves as a reference dataset.

Microbiological diagnosis of TB is carried out in separate laboratories dedicated to this task. The results of microbiological tests are sent back to the hospital or clinic which requested the test. At the same time, by law, the head of the laboratory is obliged to immediately send information about a positive result of bacteriological and molecular testing for TB to the competent sanitary inspector. Additionally, twice a year, the IGiChP requests all mycobacterial laboratories to directly deliver all positive culture, microscopy, and drug susceptibility test results from the last 6 months. Laboratories report the patient's name, sex, PESEL, home address, the address of the facility that requested the test and the date the material was received for testing, which is used by IGiChP to create the second case-based dataset used in this study, referred to as ‘laboratory data’. The National TB Register data as well as laboratory data for the year 2018 were available at IGiChP for the study purposes.

The NFZ is a centralised government institution financed by compulsory health insurance contributions, which finances health services provided to insured persons and reimburses the cost of medicines. NFZ receives notification of every person admitted to hospital or registered in an ambulatory clinic as TB patient directly from the corresponding facilities. NFZ cannot share case-based data of the patients due to legal constraints. Two aggregated datasets were requested and acquired from the NFZ for the study purposes: inventory of hospital admissions of TB patients in 2018 and inventory of TB patients registered in outpatient clinics in 2018. Table 1 summarises the datasets used for the study purposes.

**Table 1:** Characteristics of the datasets used for studying completeness of tuberculosis case notifications, Poland, 2018 (n = 3 datasets)

Data source	National TB Register data	Laboratory data	NFZ data
**Collection method**	Mandatory paper notification of TB patients from doctors, via state sanitary inspectors, to IGiChP	Mandatory notification of drug susceptibility testing results from laboratories to IGiChP	Mandatory electronic notification of TB patients admitted to hospitals or outpatient clinics
**Submission to IGiChP**	Quarterly	Twice a year	Upon request
**Case definition**	Microbiological confirmation by culture of a clinical specimen or physician's decision to recognise and treat TB based on clinical profile; only TB cases with microbiological confirmation by culture of a clinical specimen were included in the study	Laboratory confirmation of TB (microbiological confirmation by culture of a clinical specimen)	Admission to care with diagnosis of TB; only TB cases with microbiological confirmation by culture of a clinical specimen were included in the study
**Years included**	2017–2019	2017–2019	2018
**Dataset type**	Case-based	Case-based	Aggregated
**Variables extracted**	Name, surname, PESEL, address including voivodeship, age, and sex^a^	Name, surname, national identification number (PESEL), address including voivodeship, age, and sex^a^	Sex^a^, age group (0–14, 15–19, 20–44, 44–65, > 65 years), voivodeship, and ICD-10 codes A15–A19.9

ICD: International Classification of Diseases; IGiChP: National Tuberculosis and Lung Diseases Research Institute; NFZ: National Health Fund (national health insurance provider); PESEL: national personal identification number; TB: tuberculosis.

^a^ Sex information is collected as male, female, or data missing.

Both the ambulatory clinic and hospital care datasets from NFZ contained considerably higher numbers of TB patients than the National TB Register and laboratory data, with the differences particularly pronounced in certain strata of the data (e.g. hospitalisations in Kuyavian–Pomeranian voivodeship: 317 reported cases vs 197 patients notified to National TB Register; ambulatory clinic records in West-Pomeranian voivodeship: 637 reported cases vs 178 notified cases; ambulatory clinic records of female patients: 1,973 reported cases vs 1,081 notified cases; ambulatory clinic records of patients in the age group 20–44 years old: 1,738 reported cases vs 1,194 notified cases). Based upon detailed inspection in each stratum of the aggregated data (e.g. proportion of men and women in the datasets, consistency between reported data, probable duplications) and after consultation with Polish TB surveillance experts, the entirety of the ambulatory clinic dataset as well as data for three voivodeships from the hospitalisation dataset (Kuyavian–Pomeranian, Lesser Poland, and Lubusz) were deemed to have insufficient data quality and were excluded from further analysis.

### Deterministic record linkage

The case-based records of patients reported in the laboratory data were deterministically matched with the records from the National TB Register using PESEL, surname, name, and address. We first matched exclusively the records registered in both datasets in the year 2018. However, as we observed that multiple records were registered with delay in one or both registers, we conducted the matching a second time by expanding the time period: the remaining unmatched records from the National TB Register from 2018 were matched to laboratory data from the years 2017–2019, and the remaining unmatched records from the laboratory data from 2018 were matched to National TB Register from the years 2017–2019. In this manner we were able to ascertain the true number of unmatched records resulting from under-reporting (instead of from delayed notification).

### Calculating National TB Register under-reporting based on comparison of observed case-based data

The observed under-reporting was calculated as the number of unique records from the laboratory data (i.e. the records under-reported in the National TB Register), divided by the total number of records reported for 2018:

$Observed\;underreporting=\;\frac{n_{lab\;only}}{n_{lab\;only}+n_{National\;TB\;Register\;only}+n_{linked}}\times 100\%$, 

where *n_lab only_* is the number of cases reported uniquely by laboratories in 2018; *n_National TB Register_* *_only_* is the number of cases reported uniquely by National TB Register 2018; *n_linked_* is the number of cases linked between the National TB Register 2018 and laboratory data.

### Calculating National TB Register under-reporting based on number of cases estimated by two-source capture–recapture

The CRC analysis was performed using R package Rcapture [19]. Closed population models were built using the ‘closedp’ function, with the vectors representing captures of patients in National TB Register and laboratory data as arguments. The model presenting minimal Akaike’s Information Criterion was selected to estimate the total number of TB patients in Poland. CRC-based under-reporting was calculated as the number of notifications in National TB Register divided by the total CRC-modelled number of patients:

$Underreporting\;based\;on\;CRC=\;\frac{n_{National\;TB\;Register}}{n_{CRC}}\times 100\%$, 

where *n_National TB Register_* is the number of cases in National TB Register 2018; *n_CRC_* is the total number of TB cases in 2018 modelled by CRC.

### Estimating National TB register under-reporting compared to National Health Fund aggregated data

The number of patients registered in hospitals, aggregated by sex, age, and voivodeship were compared with the number of patients in the National TB Register aggregated by the same categories. TB under-reporting in the National TB Register in comparison to NFZ data was calculated as the difference between the numbers of TB cases in NFZ dataset and the corresponding strata of National TB Register, divided by the number of patients in the NFZ dataset:

$NFZ-based\;underreporting=\;\frac{n_{NFZ\;data:strata}-n_{National\;TB\;Register:strata}}{n_{NFZ\;data:strata}}\times 100\%$, 

where *n_NFZ data:strata_* is the number of cases in NFZ dataset (only included strata)*;* *n_National TB Register:strata_* is the number of cases in National TB Register (only included strata).

### Quantification of the proportion of delayed reports

The total number of patients from 2017 to 2019 who were identified in both case-based datasets was calculated. Laboratories submit the data of TB patients twice a year, and the state sanitary inspectors submit the TB notification data every quarter of a year to the IGiChP. For example, data for 2018 are to be submitted by the state sanitary inspector at the very latest in the first quarter of 2019 to be assigned to the year 2018. Therefore, a notification delay of at least 3 months may occur, which can lead to some patients not being notified in the year when TB was diagnosed, resulting in incoherence between the two case-based datasets.

Based on the deterministic record linkage of cases between the laboratory data and National TB Register we quantified (i) the number and the proportion of cases whose laboratory results were already reported in 2017 but whose notifications were submitted to IGiChP in 2018 (i.e. delayed notifications), (ii) the number and proportion of cases which were notified in 2018, but their laboratory results were only submitted in 2019 (i.e. delayed laboratory reports).

## Results

National TB Register and laboratory case-based datasets available at IGiChP contained 4,075 and 4,079 case reports from the year 2018, respectively. The datasets were verified for duplicated reports. Eleven duplications were identified in the laboratory data, resulting in 4,068 unique cases included for further analysis.

The aggregated hospital inventory dataset provided by NFZ for the year 2018 contained altogether 4,549 patients. A total of 879 patients reported for Kuyavian-Pomeranian, Lesser Poland and Lubusz (three voivodeships representing ca 16% of the country population) were excluded from the calculations, resulting in 3,670 patients from 13 voivodeships included in the analysis.

The flowchart presenting the numbers included in the calculation of under-reporting results is provided in the Supplementary Figure 1.

### Deterministic record linkage

**Figure 2:**
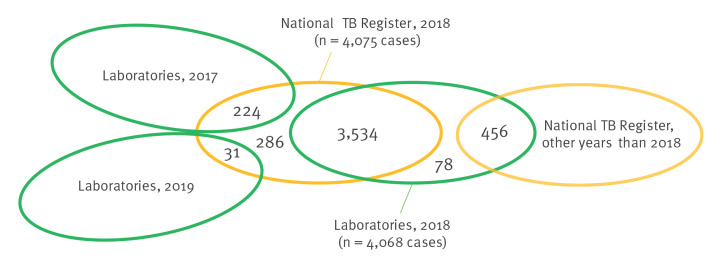
Results of deterministic record linkage between the laboratory and National TB Register datasets, Poland, 2018 (n = 4,075 TB cases in National TB Register)^a^

The results of the deterministic record linkage for the case-based datasets are presented in Figure 2.

TB: tuberculosis.

^a^ The National TB Register is the reference dataset for this study; it contained 4,075 notified TB cases in 2018.

The laboratory register contained 4,068 cases in 2018. A total of 3,534 of these cases were also reported in the National TB register on that year. Subsets of all patients notified in the National TB Register 2018 were registered by the laboratories in 2017 (n = 224) and 2019 (n = 31). A subset of all patients registered by laboratories in 2018 were notified to the National TB Register in other years than 2018 (n = 456). There were 286 patients uniquely notified in the National TB Register in 2018, and 78 patients uniquely reported in the laboratory register in 2018.

### Under-reporting based on case numbers observed or estimated by capture–recapture analysis

We calculated under-reporting from our comparison of the reported cases, and also from case numbers estimated by CRC-modelling. CRC-modelling was (i) based on the matching of records assigned exclusively to 2018 (Supplementary Table 1) and (ii) accounting for notification delay, based on the matching including data from years 2017 and 2019. We considered the second approach as correct, since some of the patients missing in the respective case-based datasets for 2018 were not under-reported, but rather reported in either 2017 or 2019.

A total of 3,789 (*n_linked_*) reported patients were linked between National TB Register 2018 and laboratory data (Table 2), including 3,534 patients linked between National TB Register 2018 and laboratory data 2018, and additional 255 patients linked between National TB Register 2018 and laboratory data from 2017 and 2019. In the laboratory data, 286 patients from the National TB Register were missing (*n_National TB Register only_*). Among patients recorded through laboratory data 2018, 534 were not notified to the National TB Register in 2018, of which 456 were notified in other years, resulting in the total of 78 patients from laboratory data not notified in National TB Register (*n_lab only_*). The observed total number of patients for 2018 was 4,153 (3,789 + 78 + 286), indicating an observed under-reporting level of 1.9% in the National TB Register (78/4,153 × 100%).

**Table 2:** Results of the record linkage between the laboratory register and National TB Register and estimation of TB under-reporting based on observed and CRC-modelled number of TB patients, Poland, 2018

Variables	Laboratory register	National TB Register	Result
Record linkage between registers			
**TB patient reported in dataset**	Yes	Yes	(a) 3,789
	Yes	No	(b) 78^a^
	No	Yes	(c) 286^b^
Calculating under-reporting based on observed case numbers			
**Total number of TB patients (d) = (a) + (b) + (c)**			(d) 4,153
**Number of TB patients notified in National TB Register (e) = (a) + (c)**			(e) 4,075
**% of total number of patients notified in National TB Register (f) = (e)/(d) × 100%**			(f) 98.1%
Observed under-reporting (g) = 100% − (f)			**(g) 1.9%**
Calculating under-reporting based on case numbers estimated by CRC analysis			
**Modelled number of patients from CRC**			(h) 4,176 (95% CI: 4,165–4,187)
**% of modelled number of patients notified in national TB Register (i) = (e)/(h) × 100%**			(i) 97.6% (95% CI: 97.3–97.8%)
CRC-based under-reporting (j) = 100% − (i)			**(j) 2.4% (95% CI: 2.2%–2.7%)**

CI: confidence interval; CRC: capture–recapture-analysis; TB: tuberculosis.

^a^ 534 cases not found in the National TB Register 2018 minus 456 cases found in the National TB register 2017, 2019.

^b^ 541 cases not found in the laboratory register 2018 minus 255 cases found in the laboratory register 2017, 2019.

The CRC modelling approach revealed a total number of patients of 4,176 ($n_{CRC}$, standard error: 5.58; 95% CI: 4,165–4,187), indicating an under-reporting level of 2.4% ((4,176 − 4,075)/4,176 × 100%; 95% CI: 2.2–2.7%) in the National TB Register.

### Under-reporting calculation based on aggregated data

**Figure 3:**
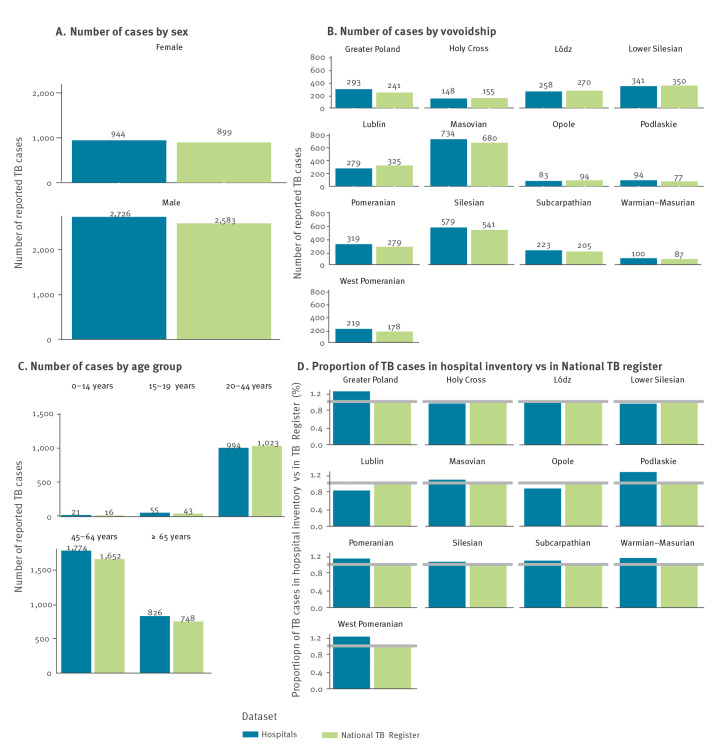
Comparison of TB case numbers in the hospitals’ inventory and national TB register, stratified by (A) sex, (B) voivodeship and (C) age group, as well as proportions of TB patients in the hospitals’ inventory vs the National TB register (D) by voivodeship, Poland, 2018 (n = 2 datasets)

The number of cases based on the hospital data was calculated for 13 of 16 Polish voivodeships, which reported in total 3,670 TB cases (*n_NFZ:strata_*) for 2018. A total of 3,482 cases were notified from those voivodeships to National TB Register (*n_National TB Register:strata_*). Completeness of the notifications in National TB Register equalled 94.9% (3,482/3,670 × 100%) and the corresponding under-reporting equalled 5.1%. The under-reporting was similar among men (5.2%; 2,583 cases in National TB Register vs 2,726 cases in NFZ data) and women (4.8%; 899 vs 944 cases) (Figure 3). The difference between the reported absolute number of cases was smallest for age group 0–14 years old (16 vs 21 cases) which nevertheless indicated high under-reporting rate for this age group (16/21, Figure 3). Calculation results in 13 voivodeships ranged from 14.2% over-reporting in Lublin (325 vs 279 cases) to 23.0% under-reporting in West-Pomeranian voivodeship (178 vs 219 cases).

TB: tuberculosis.

### Quantification of the proportion of delayed reports

Of 4,075 TB patients notified to the National TB Register in 2018, 224 (5.5%) were already registered in laboratories in 2017 and 31 (0.7%) were first registered in laboratories in 2019.

## Discussion

We estimated the completeness of notifications in the Polish National TB Register in 2018 as being over 94%. The estimation was conducted (i) via comparison of the case-based TB notifications of culture-positive cases in the Polish National TB Register and laboratory data (observed under-reporting), (ii) via two-source CRC analysis using case-based TB notifications of culture-positive cases in the National TB Register and laboratory data, as well as (iii) via calculation of the number of culture-positive patients hospitalised due to TB based on aggregated data from NFZ. The three approaches consistently indicated TB under-reporting in Poland for the year 2018 as lower than 10%: (i) 1.9% observed under-reporting, (ii) 2.4% CRC-based under-reporting, and (iii) 5.1% under-reporting comparing with the aggregated NFZ hospitalisation data. Under-reporting lower than 10% is suggested by WHO as a benchmark for a well-functioning TB surveillance system [3], which is met in Poland.

This study encountered difficulties regarding the acquisition of case-based data due to strict data protection regulations. For inventory studies, it is recommended to utilise a minimum of three independent case-based datasets [17]. However, acquisition of case-based data proves to be a repetitive problem in Europe [4-11]. In this study, as well as in studies conducted previously, healthcare providers (hospitals, insurance companies, and others) refused to share case-based data because they were either unsure whether they had the legal right to do so (even when anonymised), or the internal institutional policies prevented data sharing, and/or they lacked capacity to establish legal grounds for transfer of the data [4,9]. Hence, it is not always possible to meet the recommendations of including three independent, case-based data sources in the CRC analysis. Additionally, the assumption of a closed population structure was not met due to migration and deaths. To overcome these challenges, we used the previously suggested double-pronged inventory study design [9]. The goal of this approach is an indirect validation of the results obtained from a two-source CRC analysis. Under-reporting calculations, by comparing notifications in the National TB register to either numbers of cases estimated by CRC-modelling or to numbers of cases based on NFZ aggregated data, yielded similar results, with a difference of 2.7% between the under-reporting estimates.

An advantage of the National TB Register and laboratory data in Poland was the availability of case-based data without anonymisation or pseudonymisation. This allowed for a robust deterministic record-linkage between the datasets using name, surname, PESEL and address of the reported patient. The deterministic record-linkage indicated an overlap of 3,789 TB patients notified in 2018 with patients reported by laboratories in 2017–2019, with 3,534 of 4,075 (87%) notified patients overlapping between the two registers solely in 2018. Additional cases were identified in the laboratory data from 2017 and 2019, indicating a notification delay of more than 3 months by doctors and laboratories. Such delays in notification may lead to reporting bias and errors in annual incidence calculation since some of the diagnosed cases are notified for the following reporting year. Delayed notification of 5.5% of the patients who should be notified in 2017 indicates, that there is room for improvement in the functioning of the Polish TB notification system when it comes to timely reporting. The quantification of the proportion of delayed notifications will support evidence-based strategy adjustment of the Polish public health service directed towards improvement of timely notification. Since the TB notification system in Poland is paper-based, improvement is expected to be achieved, among others, by the already planned introduction of a digital case-based surveillance system. The latter, which will not require the time-consuming processes of sending case reports via traditional post to the state sanitary inspector and then to the IGiChP, offer a more reliable functionality, and facilitate routine data quality assurance.

Our original study protocol had called for the comparison of aggregated TB patient data from two separate data sources: hospitalisation and ambulatory care, to the National TB Register. However, the ambulatory care dataset from NFZ had to be excluded from the study due to excessively high number of reported cases, which in certain population groups exceeded the notified cases and cases reported by hospitals by more than 50%, calling into question the reliability of the ambulatory data. This problem has been previously observed in ambulatory care datasets in other inventory studies [4,20] and we speculate that the high numbers reported by outpatient clinics result from multiple registrations of the same patients who either (i) visit their doctors for different reasons after completion of their TB treatment (while remaining registered as TB patients) or (ii) visit several doctor practices in their surrounding during their disease course. Potentially, the primary suspicion of TB might also have been erroneously reported as the final diagnosis for some of the patients. A more precise patient registration process in outpatient clinics could contribute to availability of a third case-based dataset to be used for inventory studies, provided that sharing personal data from such registers for research purposes will become possible.

The NFZ hospitalisation data for all voivodeships contained 4,549 hospitalisations due to TB in 2018, 10.4% (100% − 4,075/4,549) more than the number of TB notifications for that year. However, also in this dataset there were strata of data from three voivodeships where sufficient data quality could not be assured. According to the Polish surveillance experts’ opinions, in these particular strata, a number of patients could have been registered incorrectly in the hospital inventories (e.g. patients for whom primary diagnosis of TB was not confirmed but the initial registration was not corrected, or patients who were hospitalised due to TB more than once and each hospitalisation was reported). Considering this, we calculated the level of TB under-reporting in Poland based on the NFZ hospitalisation data excluding the three voivodeships. The resulting under-reporting estimation of 5.1% for 13 of 16 voivodeships is higher than the CRC-modelled under-reporting of 2.4%, as well as than the observed under-reporting of 1.9%. Despite the small difference in the absolute numbers reported for the age group of 0–14-year-old (16 cases reported in National TB Register vs 21 in NFZ data), the high under-reporting proportion (16/21) and particular vulnerability of this group calls for more attention to correctly and completely report children and adolescents with TB.

Our study has certain limitations. First, the CRC analysis results must be interpreted with care since the analysis was performed with only two independent data sources and the assumption of a closed population was likely not met. While the latter should not have a big impact on the result of the inventory study in Poland in 2018 (most of all due to small number of immigrants with TB in the study period), the former could lead to misestimation of the TB under-reporting in both directions (i.e. underestimation, if it turned out that a certain population group is underrepresented in both National TB Register and laboratories; or overestimation, if it turned out that the only patients missing in the National TB Register are those reported in the laboratory data). Even though inventory studies are expected to result in a more accurate estimation of TB reporting completeness in comparison to other methods, it is still possible to miscalculate TB under-reporting by CRC because of potential errors in the data used, such as incorrectly written identifying information, which could prevent the same patient in two different datasets from being matched. This can lead to overestimation of under-reporting. On the other hand, inventory studies can only estimate the under-reporting of diagnosed TB, since undiagnosed cases are not listed in any TB register; this could imply that the real TB incidence in Poland is higher than estimated, despite the high diagnostic capacity. Since only cases with positive culture results can be matched between the laboratory and National TB Register datasets, we could only account for those in our under-reporting estimation. Due to lack of a second case-based dataset reporting cases without culture results, we could not verify the completeness of the notifications for the 1,412 TB patients notified in National TB Register without culture results.

In Poland, the rate of pulmonary TB is particularly high. For example, this was 95.2% in 2019 [13], compared to 77.2% in European Union/European Economic Area [21]. Since diagnosis of extrapulmonary TB is more difficult, takes longer, and extrapulmonary TB might present negative PCR or culture results, one cannot exclude the possibility that extrapulmonary TB is underdiagnosed in Poland. This could in turn mean that the true incidence of TB in Poland could be higher than the estimation resulting from our inventory study. On the other hand, the high ratio of pulmonary to extrapulmonary TB in Poland has been a stable trend over many years, which could also indicate that it is a characteristic of the population of TB patients in Poland [13,22-25]. In the future, studies based on more than two case-based datasets and/or dedicated to TB under-reporting in Poland after 2018 would help to validate our results as well as to place them in a context – for example, revealing whether TB under-reporting in Poland changes over time. Despite the high reporting completeness estimated in this study, Poland can strive for further decrease in the under-reporting of the TB cases.

The results of our TB inventory study for Poland are comparable with the results of some other inventory studies recently conducted in Europe. TB reporting completeness level was estimated with a double-pronged inventory study in Germany covering 2013–2017, as being above 90% [9]. Three-source CRC analysis indicated reporting completeness of 98.4% in Denmark, 76.5% in Finland, and 77% in Portugal in 2014–2016 [4]. For the same period, observed reporting completeness rates were 100% for Slovenia, 82% for the Netherlands, and 74% for Croatia [4]. Rates of reporting completeness estimated using other methodologies ranged from 20% in 2004–2008 in Greece and 31% in 1999–2008 in Central Italy, to more than 80% in 1999–2002 in England [5,7,26]. Despite the differences in study methodologies and findings, multiple study groups have reported similar problems as those observed in Poland: difficulties in acquiring three case-based datasets and insufficient quality of ambulatory care data.

The under-reporting estimation calculated in our study is smaller than the under-reporting estimated by the WHO for Poland for 2018 (5.1% vs 9%) [15]. This possibly results from differences in the implemented methodologies, as we used methods adjusted to country-specific TB surveillance procedures – specifically, other data sources were considered in addition to the National TB Register. Comparable differences between the results of inventory studies, which were conducted based on country-specific data sources, and the WHO estimates based on standard adjustment of case numbers for the high-income countries have been recently reported, e.g. for Denmark in 2016 (under-reporting was estimated at 1.3%, in contrast to the WHO estimate of 5.2%) [4,27] and Germany in 2017 (under-reporting was estimated at 7.0%, in contrast to the WHO estimate of 11.9%) [9,28]. According to the reporting completeness of over 90% benchmarking a good TB surveillance system coverage [3], Poland can rely on its TB notification incidence as a proxy for the true TB incidence in the country.

## Supplementary Data

146000 Supplementary Material
