# Supplementary material for: Inventory study on completeness of tuberculosis case notifications in Poland in 2018
Source: Euro Surveill. 2024 Jan 4;29(1):2300081. doi: 10.2807/1560-7917.ES.2024.29.1.2300081 (PMC10905660; doi:10.2807/1560-7917.ES.2024.29.1.2300081)
Supplement: Supplementary Material [file 2300081_SupplementaryMaterial.pdf]

This supplementary material is hosted by *Eurosurveillance* as supporting information alongside the article “Inventory study on completeness of tuberculosis case notifications in Poland in 2018”, on behalf of the authors, who remain responsible for the accuracy and appropriateness of the content. The same standards for ethics, copyright, attributions and permissions as for the article apply. Supplements are not edited by *Eurosurveillance* and the journal is not responsible for the maintenance of any links or email addresses provided therein.

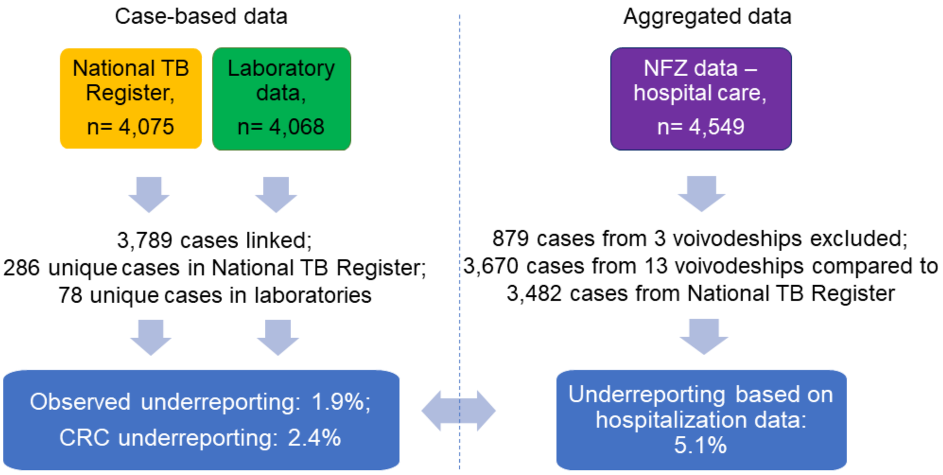

Supplementary Figure 1. Flow chart of the two pathways within the double-pronged inventory approach, which is used to estimate under-reporting in the National Tuberculosis Register, Poland, 2018, presenting the numbers of cases in each dataset, numbers of cases used to produce final results, and the underreporting results. NFZ – National Health Fund (national health insurance provider); CRC – capture-recapture analysis; TB - tuberculosis. Sex is collected as male, female or data missing

Supplementary Table 1: Underreporting based on matching of the results from year 2018

	**Laboratory register**	**National TB Register**	**Result**
**Patient report found in dataset (1= found; 0=not found)**	1	1	3,534
	1	0	534
	0	1	541
**Total number of patients**			4,609
**% of total number of patients notified in National TB Register**			88.4%
**Result observed underreporting**			11.6%
**Modeled number of patients (CRC)**			4,719
**% of modelled number of patients notified in national TB Register**			86.4%
**Result CRC-based underreporting**			13.6%
